# Molecular data aids pinworm diagnosis in night monkeys (*Aotus* spp., Primates: Aotidae) with the resurrection of a *Trypanoxyuris* species (Nematoda: Oxyuridae)

**DOI:** 10.1007/s11230-023-10134-z

**Published:** 2023-12-18

**Authors:** Brenda Solórzano-García, Andrés Link Ospina, Gerardo Pérez-Ponce de León

**Affiliations:** 1 Laboratorio de Parasitología y Medicina de la Conservación, ENES-Mérida, Universidad Nacional Autónoma de México, Tablaje Catastral N°6998, Carretera Mérida-Tetiz Km. 4.5́, C.P 97357 Municipio de Ucú, Yucatán Mexico; 2 Laboratorio de Ecología de Bosques Tropicales y Primatología, Departamento de Ciencias Biológicas, Universidad de los Andes, Cra. 1 Nº 18A-12, Bogotá, Colombia

## Abstract

Neotropical primates (Platyrrhines) are commonly parasitized by pinworm nematodes of the genus *Trypanoxyuris* Vevers, 1923. The taxonomic identity of *Trypanoxyuris* sampled in night monkeys (*Aotus* Iliger) has been rather controversial. Two species have been described, namely *T. microon* (Linstow, 1907) and *T. interlabiata* (Sandosham, 1950). The latter was synonymized with *T. microon* considering that the observed morphological differences corresponded to different developmental stages of the nematode rather than to differences between both species. Here, we used an integrative taxonomy approach, based on morphological and molecular data along with host identity, in order to assess the validity of both species. Our results evidenced that these different morphotypes correspond to different and reciprocally monophyletic groups; thus, we propose the resurrection of *T. interlabiata*. We redescribe both pinworm species using specimens sampled in *Aotus* monkeys from Colombia and discuss the advantages of combining molecular and morphological data to uncover pinworm diversity, and to understand the potential forces determining the diversification process in pinworms from platyrrhine primates.

**Supplementary Information:**

The online version contains supplementary material available at 10.1007/s11230-023-10134-z.

## Introduction

Pinworm nematodes of the genus *Trypanoxyuris* Vevers, 1923 (Oxyuridae Cobbold, 1864) are common intestinal parasites of primates (Solórzano-García & Pérez-Ponce de León, 2018; Rondón et al., 2021). They have a direct life cycle with no free-living stage and are transmitted by direct contact with food, water or surfaces contaminated with parasite eggs (Adamson, 1989); however, autoinfection and retroinfection are also routes of infection (Anderson, 2000; Felt & White, 2005; Prince, 1950). They appear to be highly host-specific with at least one species of *Trypanoxyuris* described for almost each primate genus (Hugot, 1999). Nonetheless, in those intensively studied primate species, more than one species of pinworm has been reported. Such is the case of howler monkeys, where *Alouatta palliata* Gray, *A. pigra* Lawrence and *A. seniculus* L. are each parasitized by one or two specific species of pinworms, while sharing *Trypanoxyuris minutus* Schneider, 1866 with several other species of the genus *Alouatta* Lacépède (Solórzano-García et al., 2016, 2020). Likewise, spider monkeys (*Ateles* spp. E.Geoffroy) and uakari monkeys (*Cacajao calvus* I.Geoffroy) are parasitized by two species of pinworms, *T. atelis* Cameron 1929, and *T. atelophora* Kreis, 1932 (Solórzano-García et al., 2015), and *T. cacajao* and *T. ucayalii* Conga Giese, Serra-Freira, Bowler and Mayo, 2016, respectively.

Night monkeys (*Aotus* spp.) are the only nocturnal primates in the Tropical Americas and are characterized by having a relatively small size (approximately 1 kg.), being arboreal, relying on fruits and insects in their diets. Also, night monkeys are known for living in pairs, with one adult male, one adult female and one to three offspring (Fernandez-Duque, 2007). The genus *Aotus* contains 12 recognized species distributed between Panama and Northern Argentina (Martins-Junior et al., 2022), and many of them are considered to be threatened (IUCN, 2022). Two species of *Trypanoxyuris* have been described to infect night monkeys (Travassos, 1925; Sandosham, 1950), namely *T. microon* and *T. interlabiata*, although the latter was posteriorly synonymized with *T. microon* (Inglis & Díaz-Ungría, 1960). In this study, we explore the diversity of pinworm species found in the grey-legged night monkeys (*A. griseimembra* Elliot) and the Andean night monkey (*A. lemurinus* I.Geoffroy) from two locations in Colombia. We conducted detailed morphological examinations and obtained molecular data from ribosomal and mitochondrial DNA to assess pinworm species identity and validate the existence of two monophyletic clades of *Trypanoxiuris* in the large intestine of night monkeys.

## Materials and Methods

### Specimen collection and morphological examination

Pinworm specimens were sampled from six free-ranging grey-legged night monkey individual from San Juan del Cararé, Santander Department; and one individual of Andean night monkey in Pijao, Quindio, Colombia. Pinworms were recovered from fresh monkey feces in situ and fixed in 100% ethanol. Pinworm-positive fecal samples were collected in 15 ml tubes with 70% ethanol for further examination in the laboratory for the recovery of minute male specimens (Hasegawa, 2009). To establish a linkage between morphological features and DNA sequences of parasite individuals, some specimens were cut in half with the anterior portion used for morphological exams, and the remainder posterior portion used for DNA extraction. Full body photomicrographs and measurements were taken using a Leica DM 1000 led microscope (Leica, Germany) for all the specimens before performing the cuts.

For morphological examinations pinworms were cleared with alcohol-glycerol solution and observed using an Olympus BX51 light microscope equipped with differential interference contrast (DIC). En face view observations were made using a modification of the technique proposed by Hasegawa et al. (2004), consisting of mounting the nematode inside a cut micropipette tip filled with commercial hair styling gel, attached to a microscope slide, and placing the cover slide on top (Fig. S1). The gel has the appropriate consistency for holding the nematode in vertical position allowing enface observations; gel cleanup is simple by rinsing the nematode in water, without damaging the tissue for either morphological examinations or even molecular procedures.

The saved anterior portions of the cut pinworms, as well as some complete specimens, were preserved and processed for scanning electron microscopy (SEM) by dehydration through a graded series of ethanol and then critical point dried with carbon dioxide. The specimens were mounted on metal stubs with carbon adhesive, and then gold coated and examined in a 15 kV Hitachi Stereoscan Model SU1510 scanning electron microscope. Complete specimens were deposited in the Colección Nacional de Helmintos (CNHE), Instituto de Biología, Universidad Nacional Autónoma de México (UNAM).

### DNA extraction and phylogenetic analyses

Individual pinworms were digested overnight at 56 °C in a solution containing 10 mM Tris-HCl (pH 7.6), 20 mM NaCl, 100 mM EDTA (pH 8.0), 1% Sarkosyl, and 0.1 mg/ml proteinase K. DNA was extracted using the DNAzol® reagent (Molecular Research Center, Cincinnati, OH) following manufacturer's instructions. A fragment of the mitochondrial cytochrome c oxidase subunit 1 gene (COI﻿) and a region of the large subunit of the nuclear ribosomal gene (28S) were amplified by PCR using the primers and conditions specified in Solórzano-García et al., 2020. PCR products were treated with Exo-SAP-IT (Thermo Scientific), according to the manufacturer's instructions, and sequenced at the sequencing facility of the Instituto de Biología, UNAM. All sequences obtained in this study were deposited in GenBank.

Phylogenetic relationships were assessed using Bayesian inference (BI) for each gene independently, using MrBayes v.3.2.6 (Ronquist & Huelsenbeck, 2003) and the CIPRES Science Gateway (Miller et al., 2010). Analyses were complemented with DNA sequences of nine species of *Trypanoxyuris* and three species of *Enterobius* Leach, 1853 downloaded from GenBank; *Oxyuris equi* Schrank, 1788 was included as outgroup (Table S1). Alignments of both genes were performed using MUSCLE (Edgar, 2004) through the EMBL-EBI web interface (Madeira et al., 2019). As an additional check on accuracy, COI sequences were translated into amino acids using MESQUITE v.3.2 (Maddison & Maddison, 2011), and the invertebrate mitochondrial genetic code. Most appropriate evolutionary models were inferred following the Akaike information criterion (AIC) in MrModeltest v. 2.3 (Nylander, 2004). The GTR+I+G substitution model was the best model for both genes. BI analyses included two simultaneous runs of Markov chain Monte Carlo, each for four million generations, sampling trees every 4000 generations, a heating parameter value of 0.2, and a “burn-in” of 25%. A 50% majority-rule consensus tree was constructed from the post burn-in trees. BI outputs were imported to FigTree v. 1.4 (Rambaut, 2014) for graphical visualization and editing. Genetic divergence (p-distance) between *Trypanoxyuris* from *Aotus* and the other congeneric species was calculated in Mega v.7 (Kumar et al., 2016) using the pairwise-deletion option; standard error of the distances was estimated by bootstrap resampling with 100 replicates.

**Systematics**

**Order Oxyurida**

**Family Oxyuridae Cobbold, 1864**

**Subfamily Enterobiinae Hugot et al., 1995**

**Genus** ***Trypanoxyuris*** **Vevers, 1923**

***Trypanoxyuris microon*** (**Linstow, 1907**)

Host: *Aotus griseimembra* Elliot, *A. lemurinus* I. Geoffroy

Localities: San Juan de Cararé, Santander Department; and Pijao, Quindio Departement, in Colombia.

No. Individuals: 36

Material deposited: CNHE 11896

GenBank accession numbers: COI (OR506585 – 91); 28S (OR509779 – 86)

### Redescription

**Fig 1.**
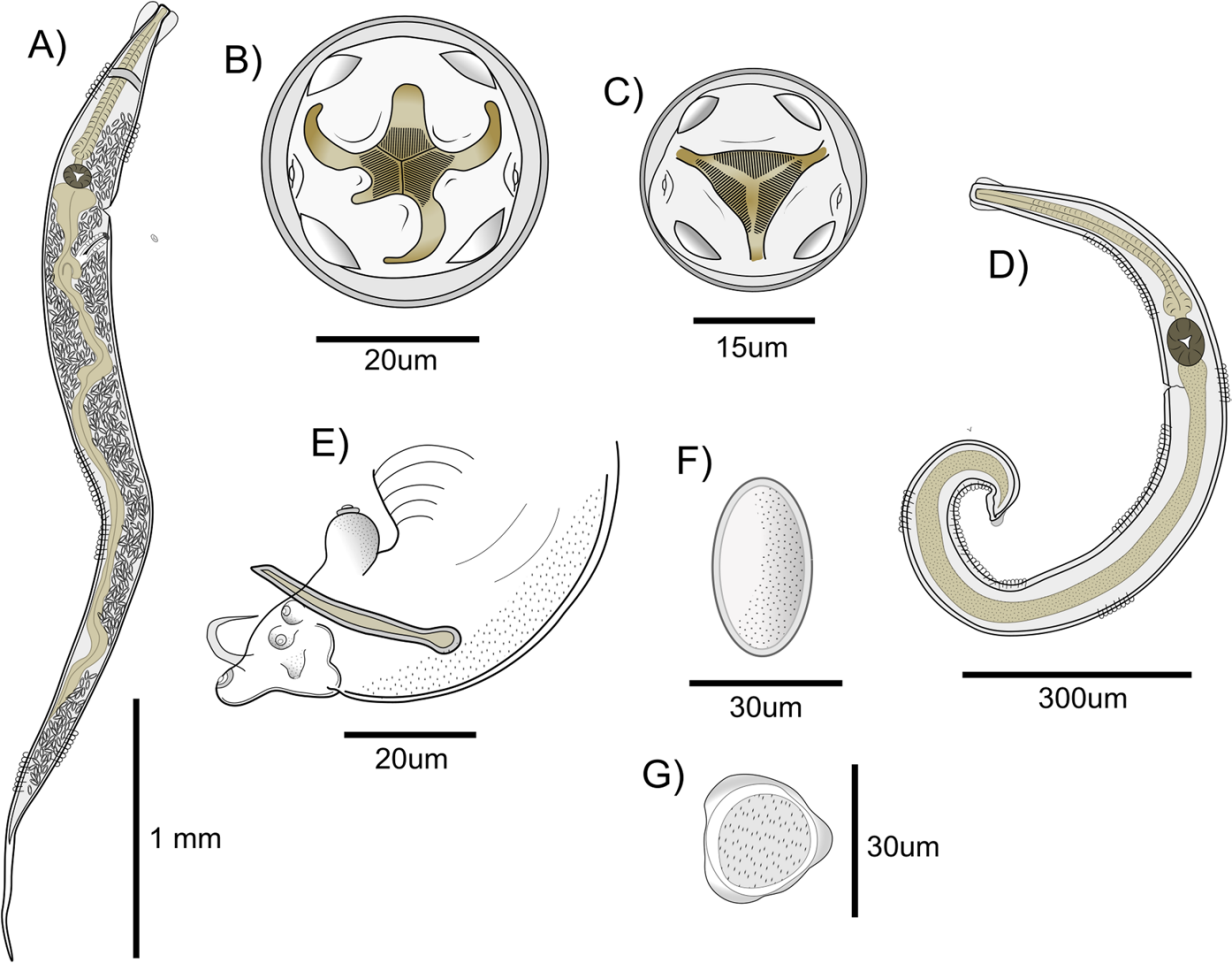
*Trypanoxyuris microon*. (A) Female full body, lateral view. (B) Female cephalic end, apical view. (C) Male cephalic end, apical view, (D) Male full body, lateral view; (E) Male posterior end, lateral view; (F) Egg; (G) Apical view of egg.

**Fig 2.**
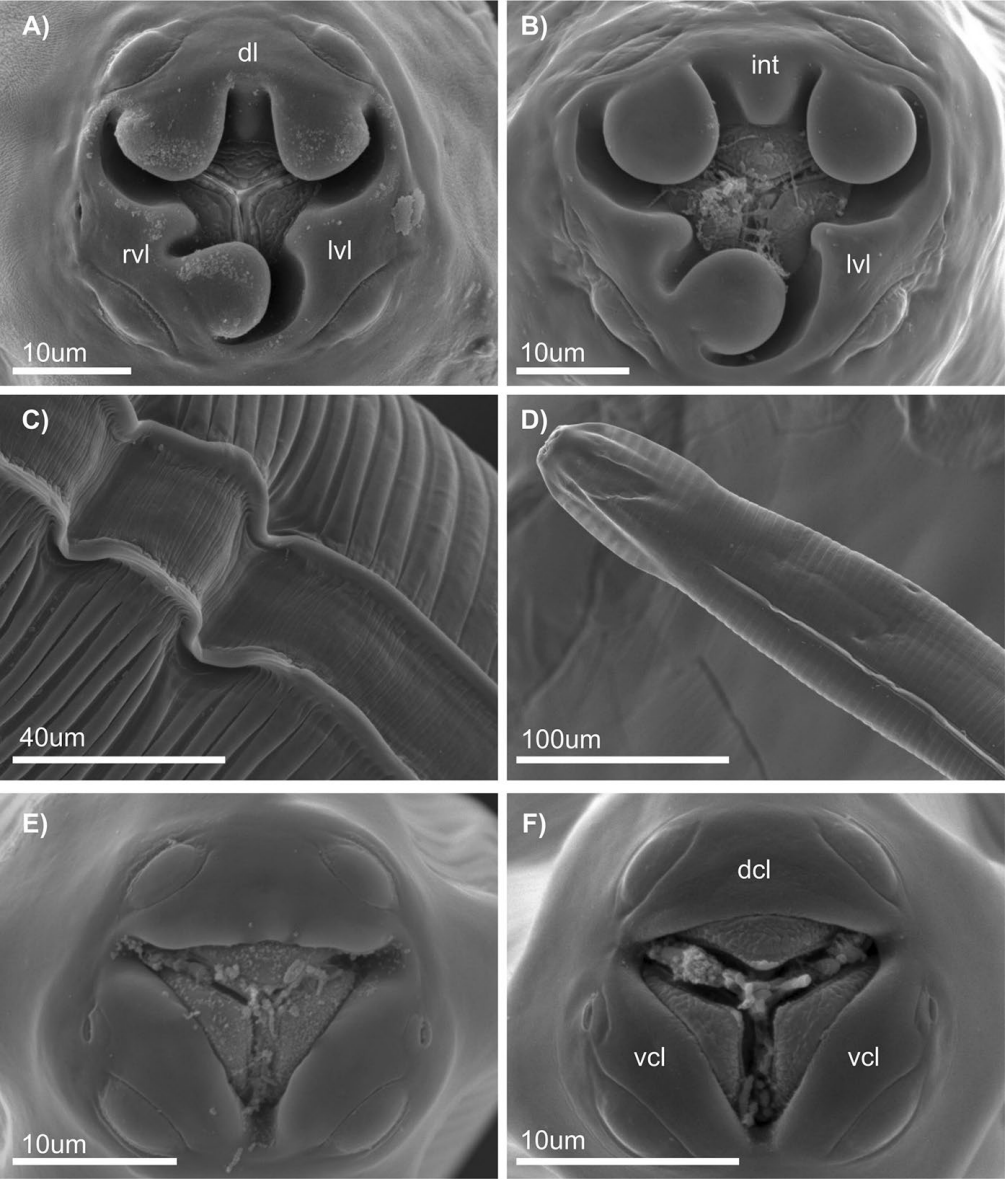
SEM pictures of pinworms found in night monkeys. *T. microon* (A, C, E); *T. interlabiata* (B, D, F). Buccal structure in females (A, B); dl: dorsal lip; rvl: right ventral lip, lvl: left ventral lip, int: interlobe. Lateral alae (C. Female, D. Male). Buccal structures in males (E, F); dcl: dorsal concave lip; vcl: ventral convex lip.

General (Figs. 1, 2A, C, D); based on 27 specimens plus six mature worms used for SEM observations, and 3 complete specimens used for DNA sequencing. White minute nematodes, females larger than males. Cuticle with transverse striations. Cephalic vesicle present. Cephalic tray circular; buccal aperture triangular, delimited by three lips, one dorsal and two subventral. Labial structures surrounded by a circular furrow (Figs. 1B, C). Cephalic papillae readily visible, located in ventral and dorsal extremes of the cephalic tray with ventral papillae closest to the amphids. Two amphids, one on each side of the cephalic tray. Lateral alae present in both sexes. Oesophagus long with posterior spherical oesophageal bulb (Figs. 1A, D).

*Females (N=17)*: Dorsal lip symmetrically bilobulated; right subventral lip asymmetrically bilobulated with upper lobe significantly smaller than lower lobe; left subventral lip reduced with a truncated inner edge (Fig. 2A). Lateral alae doble crested (Fig. 2C). Excretory pore located anterior to oesophageal bulb. Vulva located in the anterior 3rd of the body; muscular vagina longitudinally oriented, with distal vagina approximately perpendicular to longitudinal body axis. Tail long, conical. Eggs ellipsoidal, symmetric, with 3 longitudinal ridges forming a triangular contour in cross section (Fig. 1F).

*Males (N=10)*: Three unlobulated slightly squared lips, one dorsal and two subventral (Fig. 2E). Lateral alae single crested. Excretory pore located posterior to oesophageal bulb. Four pairs of caudal papillae directed ventrally, all surrounded by ring shaped thickenings; a fifth pair sessile, minute is also present directed postero-laterally (Fig. 1E). Spicule long, slightly wider in the middle. Short tail appendage.

***Trypanoxyuris interlabiata*** (**Sandosham, 1950**)

Host: *Aotus griseimembra* Elliot

Locality: San Juan de Cararé, Santander Department, in Colombia.

No. Individuals: 46

Material deposited: CNHE 11897

GenBank accession numbers: COI (OR506592 - 99); 28S (OR509772 – 77)

### Redescription

Based on 39 specimens plus six mature worms used for SEM observations, and one complete specimen used for DNA sequencing. General description follows that of *T. microon*.

**Fig 3.**
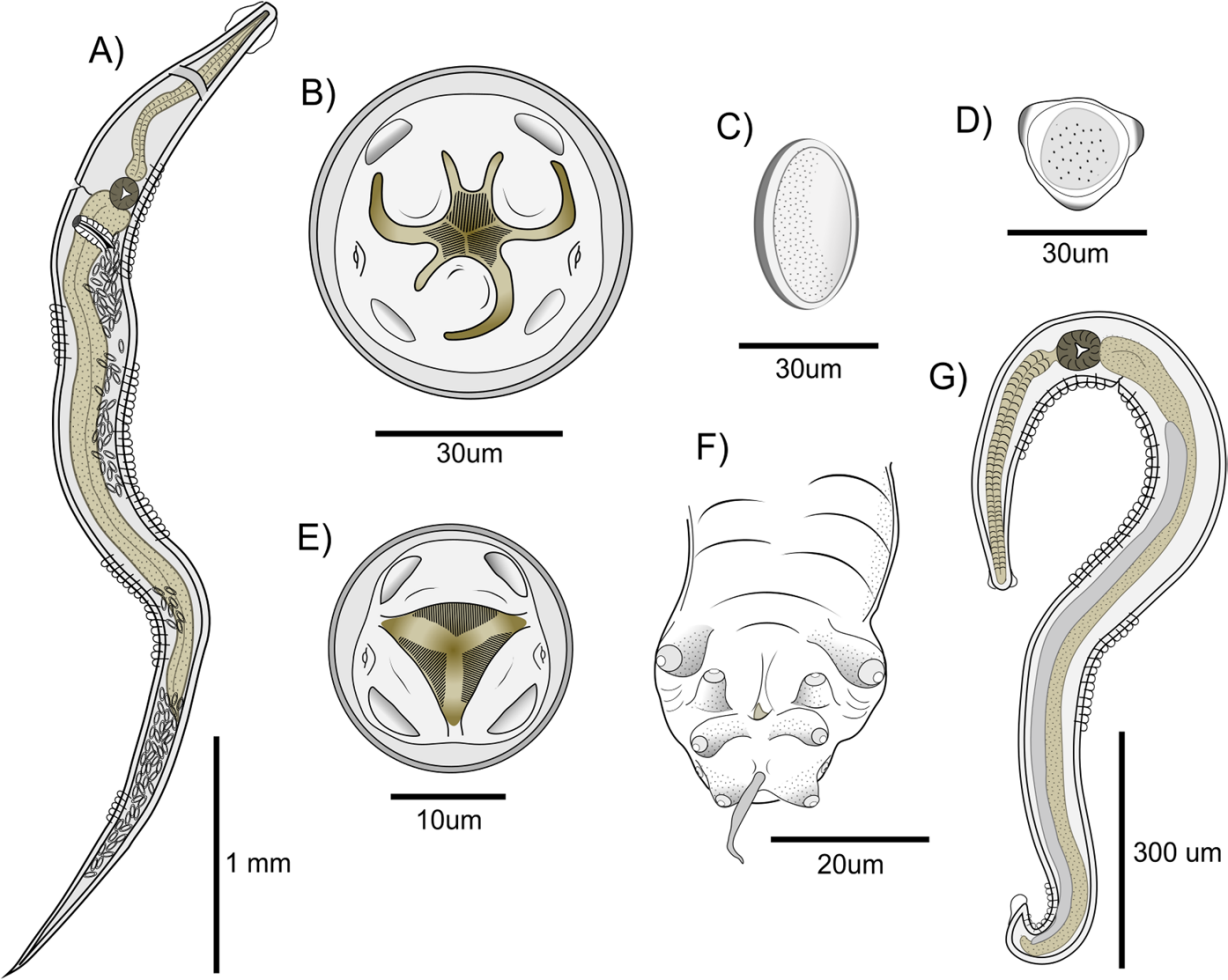
*Trypanoxyuris interlabiata.* (A) Female full body, lateral view. (B) Female cephalic end, apical view. (C) Egg; (D) Apical view of egg; (E) Male cephalic end, apical view; (F) Male posterior end, ventral view; (G) Male full body, lateral view.

*Females (N=33)*: Buccal aperture triangular; dorsal lip symmetrically bilobulated with one interlobe; right subventral lip asymmetrically bilobulated with upper lobe significantly smaller than lower lobe; left subventral lip reduced with a rounded triangular edge (Fig. 2B). Lateral alae doble crested. Excretory pore located anterior to oesophageal bulb. Vulva located in the anterior 3rd of the body; muscular vagina longitudinally oriented, with distal vagina approximately perpendicular to longitudinal body axis. Tail long, conical. Eggs ellipsoidal, symmetric, with 3 longitudinal ridges forming a triangular contour in cross section (Fig. 3C).

*Males (N=6)*: Three unlobulated lips, rounded shaped at the inner edge, one dorsal concave and two subventral convex (Fig. 2F). Lateral alae single crested (Fig. 2D). Excretory pore located posterior to oesophageal bulb. Four pairs of caudal papillae, directed ventrally, all surrounded by ring shaped thickenings; a fifth pair sessile, minute is also present directed postero-laterally (Fig. 3F). Spicule long, slightly wider in the middle. Short tail appendage.

## Remarks

Even though the morphology of these two species is very similar to each other, there are key features in the buccal structure of females that serve as diagnostic traits. Specifically, the interlobe at the dorsal lip is only present in *T. interlabiata*. Also, lips tend to have a squared shape in *T. microon,* while in *T. interlabiata* these are round shaped. Likewise, in males the inner edge of the lips tends to be square shaped in *T. microon*, and rounded in *T. interlabiata* (Fig 2E, F); however, these morphological differences between species are less conspicuous than in females making species discrimination in males a challenging task. Female buccal morphology in species of *Trypanoxyuris* from *Aotus* is peculiar in comparison with other congeners. For instance, the prominent lobes in the dorsal and right ventral lips and the reduction of the left ventral lip makes it difficult to recognize the three lips. Nonetheless, precisely these features, along with the noticeable sexual dimorphism, differentiate these *Trypanoxyuris* species from the rest. General morphology of the body, the male reproductive anatomy including the number, shape, and arrangement of caudal papillae and the presence of a spike, as well as the shape and size of the eggs are highly similar among *Trypanoxyuris* species, hence these traits are not reliable for diagnosing species. Additionally, mixed infections of *T. microon* and *T. interlabiata* were recorded in every sample of grey-legged night monkey examined, contrary to the Andean night monkey in which only *T. microon* were recorded; thus, careful enface observations are needed when characterizing pinworm diversity and infection parameters (Table 1).

## Phylogenetic analysis

**Fig 4:**
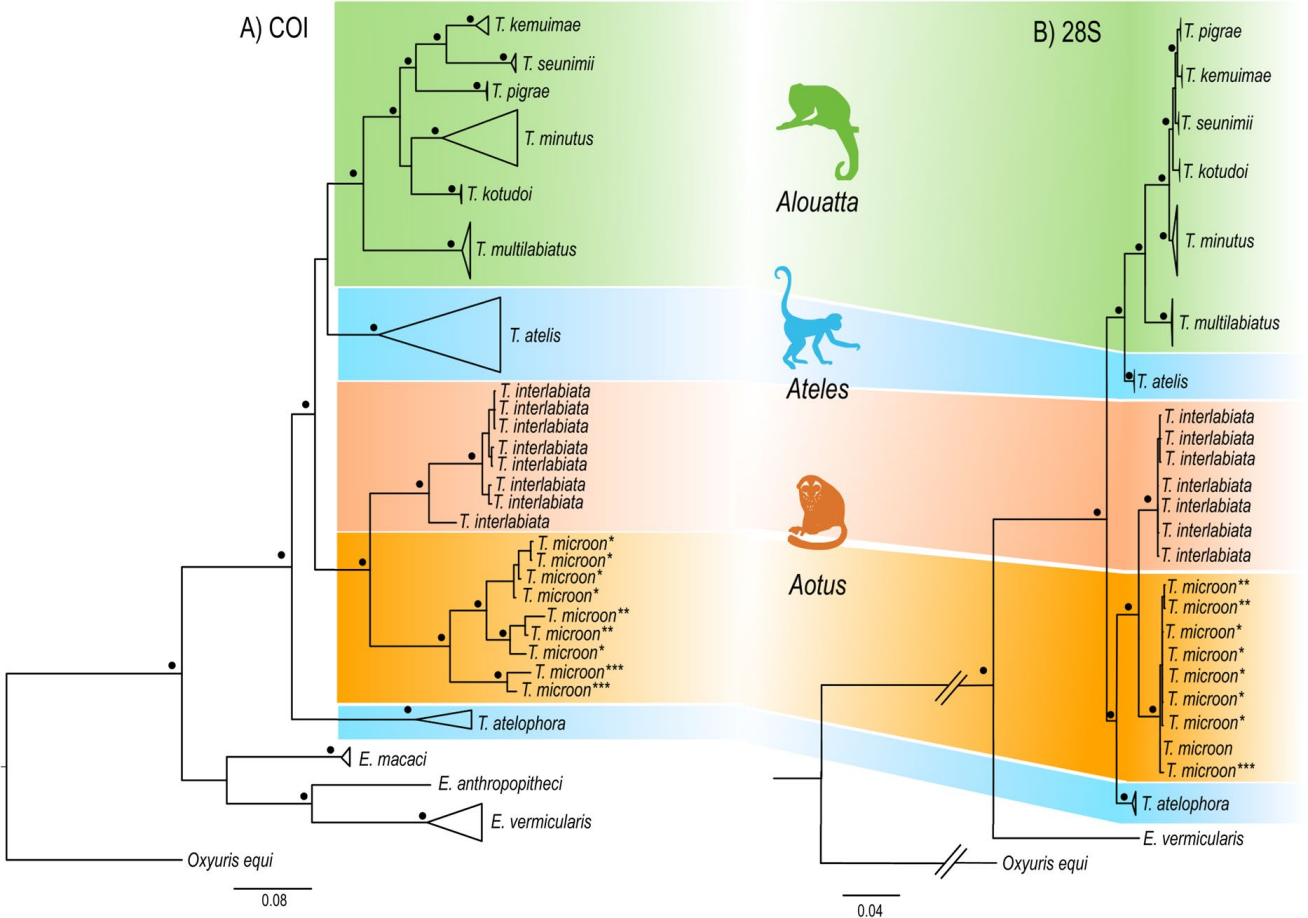
Bayesian phylogenetic tree of *Trypanoxyuris* species inferred with COI (A) and 28S (B) genes. Dots at the nodes represent posterior probability values higher than 0.95. Each color indicates a clade of *Trypanoxyuris* found in each primate genus: green – *Alouatta*; blue – *Ateles*; orange – *Aotus*. *T. microon from *Aotus griseimembra*; **T. microon from *A. lemurinus*; ***T. microon from *A. azarae* downloaded from GenBank. GenBank accessions numbers of sequences are shown in Table S1.

We obtained DNA from seven specimens of *T. microon* (two males and five females) and eight specimens of *T. interlabiata* (two males and six females). The final alignment of COI gene consisted of 47 terminals and 794 bp; while 28S alignment was 1138 bp and included 37 taxa. Phylogenetic analysis of both genes showed both species of *Trypanoxyuris* from night monkeys, *T.microon* and *T. interlabiata*, as reciprocal monophyletic groups with high posterior probability support values (Fig. 4). In the phylogenetic trees of both genes these two species are yielded as sister taxa; nonetheless, their position with respect to other *Trypanoxyuris* species is slightly different. In COI, this clade is placed as the sister group of *Trypanoxyuris* species parasitizing howler and spider monkeys, which in turn form one large clade except for *T. atelophora* which is placed as the sister species of all species (Fig. 4A). In 28S, the clade formed by *T. microon* and *T. interlabiata* formed a clade with *T. atelophora*; this clade is sister to the clade formed by *Trypanoxyuris* from howler and spider monkeys (Fig 4B). The genetic divergence values between *Trypanoxyuris* species occurring in night monkeys and the other species are shown in Table 2, with a genetic p-distance higher than 10% between *T. microon* and *T. interlabiata*. Intraspecific genetic divergence between *T. microon* from different host species range from 6.4 to 7.0% between specimens from *A. azarae* Humboldt and *A. lemurinus*, and *A. azarae* and *A. griseimembra*, respectively, and 5.1% between specimens from *A. griseimembra* and *A. lemurinus.*

**Table 1.** Measurements of adult *Trypanoxyuris microon* and *T. interlabiata* from night monkeys (*Aotus* spp.) from Colombia, and its comparison with previous descriptions. Measurements are presented in micrometres (μm) unless otherwise noted. Range followed by mean values in parentheses

Species	*T. microon*		*T. microon*		*T. microon*		*T. interlabiata*		*T. interlabiata*	
Reference	This study		Travassos, 1925		Hugot, 1985		This study		Sandosham, 1950	
Host	*A. griseimembra, A. lemurinus*		*A. trivirgatus*		*A trivirgatus*		*A. griseimembra*		*A. infulatus*	
Origin	Colombia		Brazil		Colombia		Colombia		South America	
	Female n = 17	Male n = 10	Female	Male	Female	Male	Female n = 33	Male n = 6	Female	Male
Length (mm)	3.6 – 5.2 (4.4)	1.4 – 1.5 (1.4)	4.43	1.42	5.8	1.9	3.9 – 5.4 (4.6)	1.4 – 1.6 (1.5)	5 – 6.2	2.1
Width in mid body	240.2 – 356.5 (297.0)	91.6 – 124.6 (107.0)	310	130	300	80	112.6 – 389.1 (311.9)	97.6 – 136.3 (117.0)	290 - 330	160
Nerve ring	201.7 – 264.0 (230.2)	124.7 – 151.3 (137.3)	NA	NA	220	120	203.7 – 525.4 (259.7)	119.5 – 151.4 (135.5)	170 - 180	160
Oesophagus length	674.8 – 853.8 (741.1)	336.3 – 352.9 (342.6)	730	610	920	460	842.5 – 1076.1 (927.8)	324.3 – 385.7 (355.0)	950 - 980	570
Oesophagus width at middle	30.2 – 53.7 (45.0)	22.1 – 33.8 (28.0)	NA	NA	NA	NA	36.4 – 55.6 (46.4)	25.2 – 31.9 (28.6)	50	26
Bulb length	105.4 – 127.4 (115.2)	56.1 – 72.4 (63.6)	NA	NA	100	80	99.7 – 139.7 (120.4)	52.5 – 81.7 (67.1)	NA	NA
Bulb width	103.1 – 135.8 (117.0)	54.9 – 76.8 (66.9)	NA	NA	100	70	96.7 – 139.7 (120.4)	55.1 – 89.9 (72.5)	80 – 90	50
Excretory pore – anterior end	845.0 – 1429.1 (1063.0)	400 – 444.4 (420.7)	930	NA	1100	580	819.2 – 1199.8 (1039.2)	345.3 – 458.5 (409.1)	NA	NA
Vulva – anterior end (mm)	0.85 – 1.43 (1.19)	–	1.17	–	1.5	–	0.85 -2.1 (1.3)	–	1.67 – 1.9	–
Tail length (mm)	0.81 – 1.3 (1.18)	–	1.1	–	1.0	–	0.77 – 1.3 (1.13)	–	1.5 – 1.9	–
Spicule length	–	43.6 – 48.4 (45.8)	–	49	–	46	–	43.6 – 65.8 (54.7)	–	55
Spike	–	12.3 – 22.5 (17.4)	–	NA	–	28	–	13.1 – 27.1 (20.1)	–	19
Eggs length	31.2 – 44.5 (37.6)	–	42	–	40	–	38.4 – 46.7 (43.4)	–	52	–
Eggs width	18.1 – 24.5 (21.4)	–	23	–	20	–	22.5 – 24.6 (23.4)	–	26	–

NA = not available data

**Table 2:** Average genetic divergence (percentage of p-distance) between *Trypanoxyuris* from *Aotus* and other oxyurids from primates.

	*T. microon*		*T. interlabiata*	
	COI	28S	COI	28S
*T. microon*	5.9	0.1	11.6	2.4
*T. interlabiata*	11.6	2.4	2.3	0.1
*T. atelis*	13.0	5.0	11.9	4.9
*T. atelophora*	12.9	3.6	12.5	4.2
*T. kemuimea*	11.3	6.4	11.3	6.3
*T. kotudoi*	12.4	6.1	10.5	6.3
*T. minutus*	11.8	6.2	10.9	6.2
*T. multilabiatus*	11.7	5.9	11.4	6.2
*T. pigrae*	12.2	6.3	12.2	6.2
*T. seunimii*	13.1	6.2	11.8	6.0
*Enterobius* spp.	17.2	18.7	16.7	18.5

## Discussion

The taxonomic history of *Trypanoxyuris* from *Aotus* monkeys is rather complex. The first record of *Trypanoxyuris* infecting *Aotus trivirgatus* Humboldt from Brazil was *T. microon*, which was initially described as *Oxyuris microon* Linstow, 1907. Travassos (1925) reassigned it into the genus *Enterobius* as *E. microon*. In 1950, Sandosham described a new species, *Enterobius interlabiata*, from the large intestine of *Aotus infulatus* Kuhl from a Zoological Garden in London. In his description, Sandosham mentioned the presence of three interlabia projections between the lips which are absent in other pinworm species, hence serving as the species diagnostic trait (Sandosham 1950). He also proposed that pinworms from platyrrhine primates should belong to a new subgenus named *Trypanoxyuris* which was characterized by possessing a spiked tail in males. Later, Inglis and Díaz-Ungría (1960) proposed *Trypanoxyuris* to be raised to genus level, and in the same paper, they synonymized *T. interlabiata* and *T. microon*, arguing that the observed differences in buccal structures corresponded to different developmental stages instead of characterizing different species, and thus, leaving *T. microon* as the only recognized species living in the gastrointestinal tract of night monkeys.

The results presented here corroborate the existence of these two different morphotypes in mature pinworm individuals from night monkeys. Moreover, molecular phylogenetic analyses show that these two morphotypes are well-supported reciprocally monophyletic clades. Thus, the results of this study support the existence of two different species of *Trypanoxyuris* parasitizing the genus *Aotus*. Morphologically, *T. microon* is characterized by having square shaped lips, and by lacking a dorsal lip interlobe in females. Instead, *T. interlabiata* is characterized by having round shaped lips, and a dorsal lip interlobe in females. In both species the left ventral lip in females is notably reduced and almost appears as an interlabia structure. Thus, we propose the resurrection of *T. interlabiata* as a second species of pinworm parasitizing night monkeys, appealing to the utility of an integrative taxonomic approach combining ecological, morphological and molecular information to pursue a more robust species diversity estimation. The molecular information obtained from this study expands the available genetic library, allowing accurate differentiation of *Trypanoxyuris* species from immature stages such as eggs found in feces; thus, improving the accuracy of noninvasive diagnostic procedures in primate parasitology.

Furthermore, the data presented here supports the notion of *Trypanoxyuris* as highly host specific parasites, with each genus of primates been parasitized by at least one species of pinworm (Hugot, 1999). Given previous reports on pinworm diversity and what we have observed, it seems highly plausible to postulate that more than one species of *Trypanoxyuris* is associated with each species of platyrrhine. For example, it has been proposed that *T. minutus* is shared among all species of *Alouatta*, whereas each *Alouatta* species additionally possess another particular pinworm species (Solórzano-García et al., 2016, 2020). However, for spider monkeys only two species of *Trypanoxyuris* have been reported for the whole genus (Hasegawa et al., 2004; Solórzano-García et al., 2015). In the case of *Aotus* monkeys, *T. microon* has been reported in several night monkey species including *A. azarae*, *A. trivirgatus*, *A. griseimembra*, *A. lemurinus* and *A. vociferans* (Hasegawa et al., 2012; Hugot, 1985; Michaud et al., 2003), and *T. interlabiata* has been reported in *A.infulatus*, *A. trivirgatus*, and *A. griseimembra* (Sandosham, 1950; Thatcher & Porter, 1968). A more intense effort to collect pinworms from different *Aotus* species, and to morphologically and genetically characterize them is needed to explore pinworm diversity in night monkeys, and to assess their host specificity patterns. Furthermore, the COI genetic divergence of *T. microon* sampled from different host species was higher than 5%. Reports of intraspecific genetic divergence between *Trypanoxyuris* specimens from different congeneric host species range from 6 – 10%, while divergence between linages of *Trypanoxyruis* from different host subspecies has been as low as 1.4% (Solórzano-García et al., 2019, 2020). This suggests that the *T. microon* clade maybe compose by more than one genetic linage probably related to host species. Nevertheless, more information for other *Aotus* species from different locations is still required to properly examine this hypothesis.

Currently, there are 22 genera and 171 recognized species of primates occurring in the Neotropical biogeographical region (Estrada et al., 2017), and only 24 species of *Trypanoxyuris* have been described thus far: two in Aotidae Poche, ten in Atelidae Gray, four in Callitrichidae Gray, two in Cebidae Bonaparte, and six in Pitheciidae Mivart (Hugot, 1985; Hugot et al., 1994; Conga et al., 2015; Solórzano-García et al., 2016, 2020). Given the observed patterns of more than one species of pinworm per primate genus, we could expect at least 22 new species still to be discovered. Furthermore, mixed infections seem to be common where one host individual can harbor more than one species of pinworm, and phylogenetic reconstructions and genetic assessments seem to point out towards a coevolutionary hypothesis in which pinworms and primates have shared an intimate and ancient association, showing not only a host-parasite species correspondence (Hugot, 1999), but these associations can be traced to host linage or subspecies (Solórzano-García et al., 2019) and even to host haplotypes (Solórzano-García et al., 2021), making potential pinworm diversity greater than previously expected. As we add morphological, ecological and molecular information of *Trypanoxyuris* species from different host species obtained from different locations across their range, we will contribute to a better understanding of the evolutionary and ecological dynamics that shape the associations between pinworm and primates, and the implications that diversification of one side of the interaction could have on the diversification processes on the other.

### Supplementary Information

Below is the link to the electronic supplementary material.

Supplementary file1 (PDF 21 kb)

Supplementary file2 (DOCX 17 kb)

## References

[CR1] Adamson ML (1989). Evolutionary biology of the Oxyuridae (Nematoda): Biofacies of a haplodiploid taxon. Advances in Parasitology.

[CR2] Anderson, R. C. (2000). Nematode parasites of vertebrates: Their development and transmission. Cabi Publishing.

[CR3] Conga, D. F., Giese, E. G., Serra-Freire, N. M., Bowler, M., & Mayor, P. (2015). Morphology of the oxyurid nematodes *Trypanoxyuris (T.) cacajao* n. sp. and *T. (T.) ucayalii* n. sp. from the red uakari monkey *Cacajao calvus ucayalii* in the Peruvian Amazon. *Journal of Helminthology,* 1–11. 10.1017/S0022149X1500067X 10.1017/S0022149X1500067X 26282270

[CR4] Edgar RC (2004). MUSCLE: Multiple sequence alignment with high accuracy and high throughput. Nucleic Acids Research.

[CR5] Estrada, A., Garber, P. A., Rylands, A. B., Roos, C., Fernandez-Duque, E., Di Fiore, A., et al. (2017). Impending extinction crisis of the world’s primates: Why primates matter*. Science Advances*, 1–16. 10.1126/sciadv.1600946 10.1126/sciadv.1600946 PMC5242557 28116351

[CR6] Felt SA, White CE (2005). Evaluation of a timed and repeated perianal tape test for the detection of pinworms (*Trypanoxyuris microon*) in owl monkeys (*Aotus nancymae*). Journal of Medical Primatology.

[CR7] Fernandez-Duque Eduardo, Campbell CJ, Fuentes A, MacKinnon KC, Panger M, Bearder SK (2007). Aotinae Social Monogamy in the Only Nocturnal Haplorhines. Primates in Perspective.

[CR8] Hasegawa H, Huffman MA, Chapman CA (2009). Methods of collection and identification of minute nematodes from the feces of primates, with special application to coevolutionary study of pinworms. Primate parasite ecology. The dynamics and study of host-parasite relationships.

[CR9] Hasegawa H, Ikeda Y, Dias-Aquino JJ, Fukui D (2004). Redescription of two pinworms from the black-handed spider monkey,
* Ateles geoffroyi
*
, with reestablishment of
* Oxyuronema
* and
* Buckleyenterobius
* (Nematoda: Oxyuroidea). Comparative Parasitology.

[CR10] Hasegawa H, Sato H, Torii H (2012). Redescription of *Enterobius (Enterobius) macaci* Yen, 1973 (Nematoda: Oxyuridae: Enterobiinae) based on material collected from wild Japanese macaque, *Macaca fuscata* (Primates: Cercopithecidae). Journal of Parasitology.

[CR11] Hugot, J. (1985). Sur le genre *Trypanoxyuris* (Oxyuridae, Nematoda) III. Sons-genre *Trypanoxyuris* parasite de Primates Cebidae et Atelidae. *Bulletin du Muséum National d'Histoire Naturelle, Paris, 4(1),* 131–155.

[CR12] Hugot JP (1999). Primates and their pinworm parasites: the Cameron Hypothesis revisited. Systematic Biology.

[CR13] Hugot, J. P., Morand, S., & Guerrero, R. (1994). *Trypanoxyuris croizati* n. sp. and *T. callicebi* Hugot & Vaucher, 1985 (Nematoda: Oxyuridae), two vicariant forms parasitic in *Callicebus* spp. (Primatia, Cebidae). *Systematic Parasitology, 27(1),* 35–43. 10.1007/BF02185666

[CR14] Inglis, W. G., & Díaz-Ungría, C. (1960). Una revisión del género *Trypanoxyuris* (Ascaridina: Oxyuridae). *In Nematodes de Venezuela, III* (pp. 176–212).

[CR15] IUCN. (2022). *The IUCN Red List of Threatened Species*. Version 2022-2. https://www.iucnredlist.org

[CR16] Kumar S, Stecher G, Tamura K (2016). MEGA 7: Molecular Evolutionary Genetics Analysis version 7.0 for bigger data sets. Molecular Biology and Evolution.

[CR17] Maddison, W., & Maddison, D. (2011). Mesquite: a modular system for evolutionary analysis.

[CR18] Madeira F, Park Y, Lee J, Buso N, Tamer G, Madhusoodanan N (2019). The EMBL-EBI search and sequence analysis tools APIs in 2019. Nucleic Acids Research.

[CR19] Martins-Junior, A. M. G., Sampaio, I., Silva, A., Boubli, J., Hrbek, T., Farias, I., et al. (2022). Out of the shadows: Multilocus systematics and biogeography of night monkeys suggest a Central Amazonian origin and a very recent widespread southeastward expansion in South America. *Molecular Phylogenetics and Evolution, 170.*10.1016/j.ympev.2022.107426 10.1016/j.ympev.2022.107426 35131419

[CR20] Michaud C, Tantalean M, Ique C, Montoya E, Gozalo A (2003). A survey for helminth parasites in feral New World non-human primate populations and its comparison with parasitological data from man in the region. Journal of Medical Primatology.

[CR21] Miller, M. A., Pfeiffer, W., & Schwartz, T. (2010). Creating the CIPRES Science Gateway for inference of large phylogenetic trees. *In Proceedings of the Gateway Computing Environments Workshop* (pp. 1–8). New Orleans.

[CR22] Nylander JAA (2004). MrModeltest.

[CR23] Prince MJR (1950). Studies on the life cycle of
* Syphacia obvelata
*
, a common nematode parasite of rats. Science.

[CR24] Rambaut, A. (2014). FigTree. Version 1.4.2. Edinburgh, UK.: University of Edinburgh. Tree.bio.ed.ac.uk/software/figtree/

[CR25] Rondón S, Cavallero S, Renzi E, Link A, González C, D’amelio S (2021). Parasites of free-ranging and captive American primates: A systematic review. Microorganisms.

[CR26] Ronquist F, Huelsenbeck JP (2003). MrBayes 3: Bayesian phylogenetic inference under mixed models. Bioinformatics.

[CR27] Sandosham AA (1950). On *Enterobius vermicularis* (Linnaeus, 1758) and some related species from Primates and Rodents. Journal of Helminthology.

[CR28] Solórzano-García B, Pérez-Ponce de León G (2018). Parasites of Neotropical primates: a review. International Journal of Primatology.

[CR29] Solórzano-García B, Link A, Rondón S, Pérez-Ponce de León G (2020). Pinworms of the red howler monkey (*Alouatta seniculus*) in Colombia: Gathering the pieces of the pinworm-primate puzzle. IJP: Parasites and Wildlife.

[CR30] Solórzano-García B, Melin AD, Aureli F, Pérez-Ponce de León G (2019). Unveiling patterns of genetic variation in parasite – host associations: an example with pinworms and Neotropical primates. Parasitology.

[CR31] Solórzano-García B, Nadler SA, Ponce Pérez, de León G (2015). *Trypanoxyuris atelis* and *T. atelophora* (Nematoda: Oxyuridae) in wild spider monkeys (*Ateles geoffroyi*) in tropical rain forest in Mexico: Morphological and molecular evidence. Parasitology International.

[CR32] Solórzano-García B, Nadler SA, Pérez-Ponce de León G (2016). Pinworm diversity in free-ranging howler monkeys (*Alouatta* spp.) in Mexico: Morphological and molecular evidence for two new *Trypanoxyuris* species (Nematoda: Oxyuridae). Parasitology International.

[CR33] Solórzano-García B, Vázquez-Domínguez E, Pérez-Ponce de León G, Piñero D (2021). Co-structure analysis and genetic associations reveal insights into pinworms (*Trypanoxyuris*) and primates (*Alouatta palliata*) microevolutionary dynamics. BMC Ecology and Evolution.

[CR34] Thatcher VE, Porter JA (1968). Some helminth parasites of Panamanian primates. Transactions of the American Microscopical Society.

[CR35] Travassos, L. (1925). Revisão do gênero *Enterobius* Leach, 1853. *Fauna Brasiliense. Nematodes, Oxyuroidea-Oxyuridae, (2)*, 5–11.

