# Supplementary figures and images for: Molecular data aids pinworm diagnosis in night monkeys (Aotus spp., Primates: Aotidae) with the resurrection of a Trypanoxyuris species (Nematoda: Oxyuridae)
Source: Syst Parasitol. 2023 Dec 18;101(1):1. doi: 10.1007/s11230-023-10134-z (PMC10725851; doi:10.1007/s11230-023-10134-z)

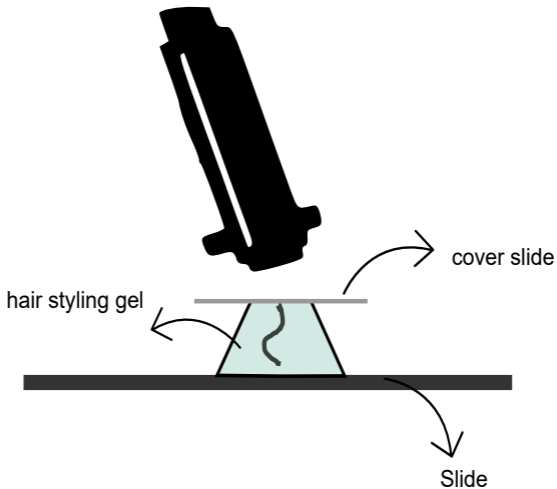

Supplement: Supplementary file 1 — Supplementary file1 (PDF 21 kb) [file 11230_2023_10134_MOESM1_ESM.pdf]
