# Supplementary material for: Molecular data aids pinworm diagnosis in night monkeys (Aotus spp., Primates: Aotidae) with the resurrection of a Trypanoxyuris species (Nematoda: Oxyuridae)
Source: Syst Parasitol. 2023 Dec 18;101(1):1. doi: 10.1007/s11230-023-10134-z (PMC10725851; doi:10.1007/s11230-023-10134-z)
Supplement: Supplementary file 2 — Supplementary file2 (DOCX 17 kb) [file 11230_2023_10134_MOESM2_ESM.docx]

**Table S1**. GenBank accession numbers of DNA sequences employed in the phylogenetic analysis of *Trypanoxyuris* species. Bold font indicates sequences obtained in this study.

| Species | Host | Country | GenBank accession numbers | |
| --- | --- | --- | --- | --- |
|  |  |  | COI | 28S |
| *Trypanoxyuris kemuimae* | *Alouatta seniculus* | Colombia | MN443910, MN443892 | MN447700-01 |
| *Trypanoxyuris seunimii* | *Alouatta seniculus* | Colombia | MN443898-99 | MN447704-05 |
| *Trypanoxyuris pigrae* | *Alouatta pigra* | Mexico | MN443890-91 | KU285468-69 |
| *Trypanoxyuris minutus* | *Alouatta palliata* | Nicaragua | MH733439 | MH733401 |
|  |  | Mexico | KU285480 | KU285467 |
|  |  | Costa Rica | MH733437 | MH733397 |
|  | *Alouatta seniculus* | Colombia | MN443901 | MN447707 |
| *Trypanoxyuris kotudoi* | *Alouatta seniculus* | Colombia | MN443903-04 | MN447711-12 |
| *Trypanoxyuris multilabiatus* | *Alouatta palliata* | Costa Rica | MH733404, MH733415 | MH733403 |
|  |  | Mexico | KU285487 | KU285470 |
|  |  | Nicaragua | MH733405 | MH733405 |
| *Trypanoxyuris atelis* | *Ateles geoffroyi* | Mexico | KU285497 | KU285474 |
|  |  | Costa Rica | MH733425 | MH733406 |
|  |  | Japan | AB626875 | ----------------- |
|  | *Ateles belzebuth* | Japan | AB626876 | ----------------- |
|  | *Lagothrix lagotricha* | Japan | AB626877 | ----------------- |
| *Trypanoxyuris atelophora* | *Ateles geoffroyi* | Mexico | KU285499 | KU285478 |
|  |  | Costa Rica | MH733431 | MH733408 |
| ***Trypanoxyuris microon*** | ***Aotus griseimembra*** | **Colombia** | **OR506585 – 88, 91** | **OR509779 – 83, 86** |
|  | ***Aotus lemurinus*** | **Colombia** | **OR506589 – 90** | **OR509784 – 85** |
|  | *Aotus azarae* | Japan | AB626878-79 | LC416070 |
| ***Trypanoxyuris interlabiata*** | ***Aotus griseimembra*** | **Colombia** | **OR506592 - 99** | **OR509772 – 77** |
| *Enterobius macaci* | *Macaca fuscata* | Japan | AB626858-59 | ----------------- |
| *Enterobius anthropopitheci* | *Pan troglodytes* | Japan | AB626860 | ----------------- |
| *Enterobius vermicularis* | *Homo sapiens* | Japan | AB626865, 68 | ----------------- |
|  | *Pan troglodytes* |  | AB626880 | ----------------- |
